# Characterization and Follow-Up of *Trypanosoma cruzi* Natural Populations Refractory to Etiological Chemotherapy in Oral Chagas Disease Patients

**DOI:** 10.3389/fcimb.2021.665063

**Published:** 2021-04-28

**Authors:** Arturo Muñoz-Calderón, Zoraida Díaz-Bello, Belkisyolé Alarcón de Noya, Oscar O. Noya-González, Alejandro G. Schijman

**Affiliations:** ^1^ Laboratorio de Biología Molecular de la Enfermedad de Chagas, Instituto de Investigaciones en Ingeniería Genética y Biología Molecular (INGEBI), Consejo Nacional de Investigaciones Científicas y Técnicas (CONICET), Buenos Aires, Argentina; ^2^ Instituto de Medicina Tropical “Dr Félix Pifano”, Universidad Central de Venezuela, Caracas, Venezuela

**Keywords:** *Trypanosoma cruzi*, Oral Chagas disease, trypanocidal chemotherapy, therapeutic failure, genetic diversity, restriction fragment length polymorphism, parasite load, minicircle signature

## Abstract

We aimed to characterize the genetic constitution of natural *T. cruzi* populations involved in an Oral Chagas Disease (OCD) outbreak at a rural school of the community of Chichiriviche de la Costa, Venezuela, which affected patients did not respond to the etiological treatment. Peripheral blood samples and/or hemocultures were obtained from twenty-nine OCD patients at time of diagnosis or along nine years of Post-treatment (Tx) follow-up. The IgG serology, *T. cruzi* discrete typing units (DTU), satellite DNA-qPCR parasitic loads, and minicircle signatures were determined at Pre-Tx and after Tx. The serological titles and parasitic loads changed after treatment, with a significant decrease of IgG titers (Spearman’s r value= -0.961) and median parasite loads from 2.869 [IQR = 2.113 to 3.720] to 0.105 [IQR = -1.147 to 1.761] log10 par eq. /mL at Pre-Tx and Post-Tx, respectively, suggesting infection evolution from acute to chronic phase, without seroconversion or parasitological eradication, which was indicative of treatment failure. All patients were infected with *T. cruzi* DTU I populations. At Pre-Tx their median Jaccard genetic distances were 0.775 [IQR = 0.708 to 0.882], decreasing in genetic variability towards the end of follow-up (Mann-Whitney U test p= 0.0031). Interestingly, no Post-Tx minicircle signature was identical to its Pre-Tx counterpart population in a same patient, revealing selection of parasite subpopulations between the primary infection and Post-Tx. The parasitic populations isolated from hemocultures showed a lower number of bands in the minicircle signatures with respect to the signatures obtained directly from the patients’ blood samples, demonstrating a process of parasitic selection and reduction of the population variability that initially infected the patients. Decrease of parasitic loads after treatment as well as Pre- and Post-Tx intra-TcI diversity might be a consequence of both, natural evolution of the acute infection to the chronic phase and persistence of refractory populations due to Tx selection.

## Introduction

Chagas disease (CD), caused by *Trypanosoma cruzi* affects mostly populations living in poor housing conditions in Latin America (Pereiro, 2019). Although initially considered transmitted mostly by triatomine bugs, the parasite can be transmitted by other routes, one of the most important in recent decades being oral transmission, as a result of contamination of food with feces of wild triatomines or secretions from reservoirs in endemic regions (Rueda et al., 2014; Alarcón de Noya et al., 2015).

Venezuela is the third country with oral Chagas disease (OCD) reports since 2007, and the first one with the most numerous OCD micro-epidemics (Alarcón de Noya et al., 2015). The first outbreaks were associated with consumption of guava (*Psidium guajava*) juice in an urban school in the Chacao Municipality (Caracas, Capital District) in 2007 (Alarcon de Noya et al., 2010) and the second in a rural school in the community of Chichiriviche de la Costa (Vargas State) (Alarcon de Noya et al., 2016) in 2009, with varied degree of disease severity and mortality (Alarcón de Noya et al., 2015; Alarcon de Noya et al., 2016).

In Chichiriviche de la Costa, a small tourist town nestled between mountains and on the shores of the Caribbean Sea, located on the north-central coast of Venezuela, there were simultaneous cases of fever and myocarditis in students, teachers, and administrative staff of the local school (10°31′53.97″N - 67°15′36.02″W). These clinical findings led to the serological screening of specific anti-*T. cruzi* IgM and IgG reactivities to 441 people, resulting in 89 infected people and mortality associated with acute *T. cruzi* infection in 5.6% of them (Alarcón de Noya and Martinez, 2009; Alarcon de Noya et al., 2016).

In these patients, the outcome observed by serological, parasitological and PCR-based monitoring two years after treatment with Benznidazole (Bnz) was disappointing. Around 70% of patients still had positive lytic antibodies; some presented anti-*T.cruzi* IgG antibody titers and positive *T. cruzi* PCR findings (Alarcón de Noya et al., 2011). Considering that Bnz is highly effective in the acute phase and during childhood in other endemic regions where other parasite DTUs prevail (Bianchi et al., 2015; Moscatelli et al., 2019), these data suggested therapeutic failure and consequently a second treatment was administered to people with persistence of lytic antibodies and/or positive PCR results, however without outcome improvement (Garcia-Bournissen, 2019; Molina-Morant et al., 2020).

It is crucial to carry out genetic studies on parasitic populations involved in OCD aiming to seek an explanation for the differences in Bnz efficacy with respect to other epidemiological settings. In assessing drug efficacy, *in vitro* susceptibility tests carried out in parasite isolates obtained at pre-treatment (Pre-Tx) and Post-treatment (Post-Tx) from these outbreaks displayed clones naturally less susceptible to Bnz and Nfx (Muñoz-Calderón et al., 2012; Muñoz-Calderón et al., 2019). Indeed, the genetic polymorphism of *T. cruzi* may be involved in its susceptibility to anti-parasitic drugs. However, genetic studies of natural parasite populations in CD patients exhibiting treatment failure have not been done so far. In this context, peripheral blood samples and hemocultures obtained from OCD patients at Chichiriviche de la Costa outbreak were analyzed at time of diagnosis and after Tx, attempting to detect and characterize fluctuations of *T. cruzi* genetic diversity and parasitic loads along the follow-up.

## Methods

### Ethics Statement

The study was approved by the Ethical Review Board of the Instituto de Medicina Tropical “Dr Félix Pifano”-Universidad Central de Venezuela, Caracas, Venezuela (CEC-IMT 019/2010 - December 10, 2010); following the principles expressed in the Declaration of Helsinki.

Written informed consent forms were signed by each participant or from their legal guardians (minor subjects were included). Samples were anonymized before being processed.

### Patients and Samples

The study population consisted of students, teachers, school workers and outsiders involved in the preparation or transportation of food consumed in schools, and anyone considered as a potential at-risk “school contact”. The participants eligible for the study were 29 patients who presented positive anti-*T. cruzi* IgG antibody titers or kinetoplastid DNA-PCR amplification after nine years of Post-Tx follow-up, according to data provided by the Instituto de Medicina Tropical “Dr Félix Pifano”- Universidad Central de Venezuela, Caracas, Venezuela.

**Figure 1:**
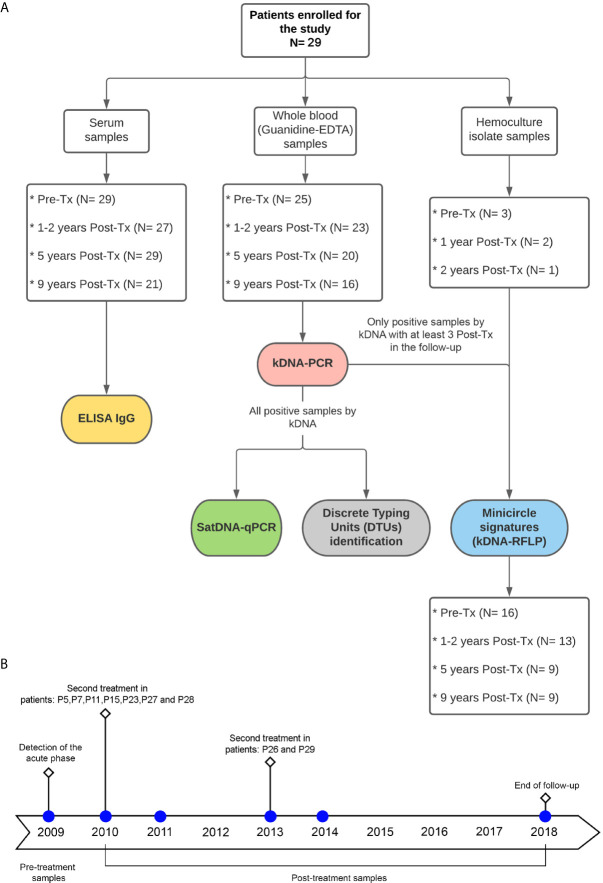
**(A)** Flowchart for the distribution of study patients´ samples and type of analysis performed. Tx: Treatment. **(B)** timeline of blood sample collection at follow-up time.

**Table 1** shows epidemiological features of the study patients from the Chichiriviche de la Costa outbreak, hemocultures obtained, number of blood samples analyzed and Post-Tx time period of each sample. Additionally, the distribution of the number of samples for each of the variables studied in this work (Pre-treatment and Post-treatment points), is outlined in **Figure 1**.

**Table 1:** Description of the Oral Chagas Disease patients´ cohort with Pre and Post treatment follow-up samples for 9 years.

Code	Origin of *T. cruzi* gDNA	Age (years)	Gender	DTU	Pre-treatment clinical status	Primary Treatment	Secondary Treatment	Number of Post-treatment samples evaluated	Culture isolate period
P1	Blood	10	F	TcI	Severe	Bnz		5	N/A
P2	Blood	7	F	TcI	Severe	Bnz		5	N/A
P3	Blood	8	F	TcI	Moderate	Bnz		4	N/A
P4	Blood	9	F	TcI	Severe	Bnz		3	N/A
P5	Blood	9	F	TcI	Severe	Bnz	Bnz^+^	5	N/A
P6	Blood	7	F	TcI	Severe	Bnz		4	N/A
P7	Blood/Hemoculture	8	M	TcI	Moderate	Bnz	Bnz^+^	5	Pre-Tx
P8	Blood	10	F	TcI	Severe	Bnz		4	N/A
P9	Blood	9	F	TcI	Severe	Bnz		4	N/A
P10	Blood	10	M	TcI	Moderate	Bnz		4	N/A
P11	Blood	10	M	TcI	Severe	Bnz	Bnz^+^	4	N/A
P12	Blood	9	M	TcI	Severe	Bnz		4	N/A
P13	Blood	6	M	TcI	Severe	Bnz		4	N/A
P14	Blood	7	M	TcI	Severe	Bnz		3	N/A
P15	Blood	7	M	TcI	Severe	Bnz	Bnz^+^	4	N/A
P16	Blood	8	M	TcI	Severe	Bnz		4	N/A
P17	Blood	9	M	TcI	Severe	Bnz		4	N/A
P18	Blood	11	F	TcI	Severe	Bnz		4	N/A
P19	Blood	16	M	TcI	Severe	Bnz		4	N/A
P20	Blood	11	F	TcI	Severe	Bnz		4	N/A
P21	Blood/Hemoculture	36	M	TcI	Severe	Bnz		4	Pre-Tx
P22	Blood	11	F	TcI	Severe	Bnz		4	N/A
P23	Blood	47	F	TcI	Severe	Bnz	Bnz^+^	4	N/A
P24	Blood	11	M	TcI	Severe	Bnz		4	N/A
P25	Blood	11	M	TcI	Severe	Bnz		4	N/A
P26	Hemoculture	5	F	TcI	Severe	Bnz	Bnz^*^	N/A	Pre-Tx
P27	Hemoculture	9	M	TcI	Severe	Bnz	Bnz^+^	N/A	1 year Post-Tx
P28	Hemoculture	10	M	TcI	Severe	Bnz	Bnz^+^	N/A	1 year Post-Tx
P29	Hemoculture	7	F	TcI	Severe	Bnz	Bnz^*^	N/A	2 years Post-Tx

Bnz^+^ - Second treatment with Benznidazole given 1 year post infection.

Bnz^++^ - Second treatment with Benznidazole given 4 years post infection.

N/A, Not Applicable.

Bzn, Benznidazole.

### Clinical Classification

Patients with acute OCD were clinically classified with i) moderate symptoms whose clinical situation allowed them to continue with work activities (facial edema; dyspnea; abdominal pain; headache; arthralgia; myalgia; asthenia; drowsiness; headache; intraocular pain; rash; echocardiogram (ECHO): Supraventricular hypertrophy), and ii) severe symptoms who had to be hospitalized or deceased (ECHO: pericardial effusion or myocarditis; electrocardiogram (EKG): supraventricular arrhythmia or supraventricular hypertrophy; adenomegaly; cardiomegaly; tachycardia).

### Antiparasitic Treatment

Patients were treated with Bnz (6 mg/kg/day) during 60 continuous days in three daily doses (Alarcón de Noya et al., 2011; Alarcon de Noya et al., 2016). During follow-up, nine treated patients who remained with evidence of parasite persistence (high values ​​in anti-*T. cruzi* IgG titers, presence of lytic antibodies, and/or persistence of positive PCR) received a new course of treatment with Bnz (Amato Neto, 1999).

### Laboratory Diagnosis

At each visit, a single peripheral blood sample was withdrawn by venipuncture. Five milliliters were dedicated to serum collection to perform IgG-based serological analyses. The remaining volume was used for molecular biology studies.

### Immunoenzymatic Assay

An in-house immunoenzymatic assay (ELISA) was performed using a delipidized antigenic lysate obtained from the epimastigote stage of the *T. cruzi “*PM” strain (TcI) and processed using Maxisorp plates. ELISA tests involving anti-human IgG alkaline phosphatase conjugates were carried out simultaneously in all sera. ELISA IgG cut-off line was defined as an optical density (OD) value equal to 0.200 to determine a positive sample (Alarcón de Noya et al., 2011).

### Preparation of Samples for Molecular Biology Studies

Five mL of patients’ peripheral blood was mixed with an equal volume of 6M Guanidine HCl/0.2 mM EDTA, pH 8.00 (GEB) and boiled according to Diaz-Bello et al. (2008). The GEB mixture was stored at 4°C; DNA was extracted from 300 µL aliquots using the High Pure PCR Template Preparation kit (Roche Diagnostics, Indianapolis, IN) and eluted in 100 µL elution buffer (Duffy et al., 2013).

### Parasite Cultures

Epimastigote forms obtained from patients’ hemocultures and four TcI reference strains (Dm28c, SilvioX10, gal4, and CMA) were cultured in LIT medium (supplemented with 5% bovine fetal serum) and sub-cultured every 15 days during the exponential growth phase.

### DNA Extraction and Quantitative Analysis

Parasites were collected at logarithmic phase and centrifuged at 3000 × *g*. The High Pure PCR Template Preparation kit (Roche Diagnostic, Indianapolis, IN) was used for DNA extraction following the manufacturer’s recommendations. The genomic DNA was then preserved at 4°C until use. DNA purity and concentration were determined in a Nanodrop ND-1000 (NanoDrop Technologies, Houston, TX, USA) at 260/280 nm wavelengths.

### kDNA-PCR Amplification

A 330 bp sequence belonging to the hypervariable region of *T. cruzi* kDNA was amplified as reported (Schijman et al., 2011). Briefly, the master mix was composed by 1X Taq platinum amplification buffer, 200 µM dNTPs, 3 mM MgCl_2_ solution, 1.5 U Taq Platinum (Invitrogen, Brazil), 10 µM kDNA specific primers 121 (AAATAATGTACGGGKGAGATGCATGA) and 122 (GGTTCGATTGGGGTTGGTGTAATATA), 5 µl of template DNA, and a quantity of water sufficient to give a final volume of 50 µl. Cycling parameters were one step of 3 min denaturation at 94°C; 2 cycles of 1 min at 97.5°C, 2 min at 64°C; 33 cycles of 1 min at 94°C, 1 min at 62°C and one final extension step of 10 min at 72°C. The kDNA-PCR products were analyzed in 2% agarose gels stained with ethidium bromide.

### Estimation of Parasitic Loads

A standardized duplex quantitative real-time PCR (qPCR) targeted to *T. cruzi* Satellite (Sat) DNA and Internal Amplification control (IAC) was used, under the reported conditions (Duffy et al., 2013). Standard curves were plotted with 1/10 serial dilutions of total DNA obtained from the same stock of a GEB seronegative sample spiked with 1x10^5^ par.eq./mL of Silvio X10 (TcI) cultured epimastigotes. The reportable linear range of this qPCR was 1 to 6 log_10_par eq./mL for TcI-infected samples (Duffy et al., 2013).

### Identification of *Trypanosoma cruzi* Discrete Typing Units (DTUs)

A Real-Time PCR-based algorithm for identification of *T. cruzi* DTUs was done by means of amplification of spliced-leader genes, 24Sα rRNA, 18Sα rRNA and COII genomic markers using TaqMan probes, as described (Cura et al., 2015).

### Minicircle Signatures

To identify minicircle signatures (Ms) of bloodstream parasite populations, kDNA-PCR samples were studied. The 330 bp purified amplicon was subjected to HindFI, RsaI, and MspI digestions as reported (Burgos et al., 2010). Signatures were visualized and digitized using a Syngene ultraviolet light transilluminator (Synoptics Ltd., Cambridge, UK), at 498 nm. The sizes of the bands of each signature were determined by comparison with molecular weight markers included in each electrophoresis, using the GelAnalyzer v2010a program (http://www.gelanalyzer.com). To assess the reproducibility of the Ms, three independent kDNA-PCR experiments from a same DNA preparation were performed, and each product was digested on triplicate.

### Genetic Distance Determination

Binary matrices were created from each Ms. Only sharp bands resolved by gel electrophoresis were used in the analysis. Visible amplified bands were scored as “1”, whereas the absence of bands of the same molecular weight was scored as “0”. All samples from a same patient were analyzed in a same gel. The degree of similarity, i.e. frequency of co-occurrence, between clinical samples was measured using the Jaccard’s coefficient (JC) between Pre-Tx and Post-Tx samples for each patient.

In order to estimate the overall variability of the Pre-Tx parasite populations using as marker the minicircle signatures, a median value of the Jaccard genetic distances among the pre-Tx samples was determined, and the profiles obtained from each sample at the different Post-Tx points were compared against that pre-Tx median value.

In addition, the minicircle profiles obtained from the hemocultures were compared among them, in order to obtain an overall JC sight of the cultured parasite strains and compare their JCs with those obtained among laboratory reference clones belonging to Tc I.

### Statistical Analysis

A data analysis was performed on the follow-up panel to evaluate the change in parasite loads, IgG antibody values, Jaccard’s coefficient, and/or the clinical classification of the patients. The graphic comparisons between the different variables obtained from each patient: IgG serology (optical density), parasite load (Par.eq/mL) and JC, were carried out with a Z score data transformation. The continuous measurements were expressed and plotted as the median and interquartile range of 25–75%, whereas dichotomous variables were expressed and plotted as a percentage.

For all variables, the normal distributions were evaluated using the Shapiro-Wilks test. The Spearman’s rank correlation coefficient was calculated to assess correlations among the variables under study (parasitemia levels, ELISA IgG values and patients’ clinical classification). Correlation is stronger as the value of *r* approaches 1 for positive correlations or -1 for negative correlations. To evaluate the differences between Pre-Tx and Post-Tx follow-up points, the Mann-Whitney U test was used.

All analyses were performed with the RStudio Team software (2020), and calculations were determined with a *p*-value of <0.05 for significant statistical differences.

## Results

Peripheral blood samples and/or hemocultures obtained from 29 OCD patients at Pre-Tx and/or at different Post-Tx visits were included in the study (**Table 1**). From each patient (except P26 to P29, in which only hemocultures were obtained), one Pre-Tx sample and Post-Tx samples collected at at least two different visits were analyzed by serological and molecular tools.

### IgG-based Serological Findings and Parasitic Loads

**Figure 2:**
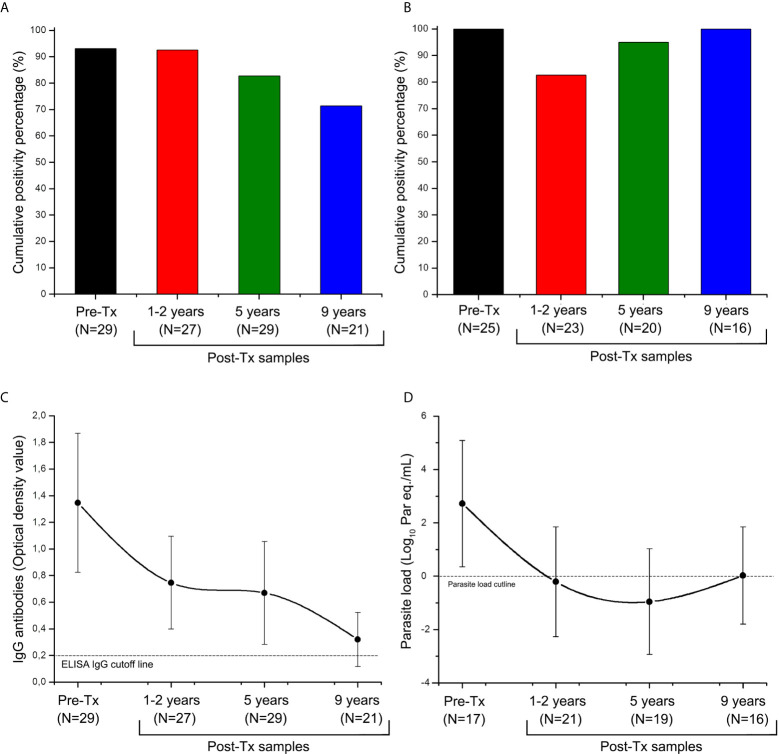
Distribution of anti-*Trypanosoma cruzi* ELISA-IgG reactivity and SatDNA-qPCR positivity during the follow-up of patients of the Chichiriviche de la Costa oral Chagas disease outbreak. **(A)** Cumulative positivity percentage of ELISA-IgG; **(B)** satDNA-qPCR cumulative positivity percentage; **(C)** Trend of optical density values for the ELISA-IgG; **(D)** Trend of parasitic load values for the satDNA-qPCR. Tx: Treatment. ELISA IgG cutline: Optical Density (OD) value reportable by a positive sample (Diaz-Bello et al., 2008); TcI load cutline: 1 par eq./mL, lowest value of linear reportable range of qPCR (Duffy et al., 2013). The data in Figures **(C)** and **(D)** are presented as median and interquartile range from 25 to 75%.

All study patients were seropositive at Pre-Tx. The Post-Tx accumulated seropositivity was 82.26% (n = 21) (**Figure 2A**). After nine years of Post-Tx, seropositivity was 71.43% (n=15), with a median in optical density of 0.320 [IQR = 0.174 to 0.507]. Although there was not seroconversion, a trend towards antibodies titers decrease was observed (**Figure 2C**).

Out of the 29 study patients, whole blood samples were collected in 25. In patient P7 and P21 whole blood and a Pre-Tx hemoculture isolate were obtained. In patients P26 to P29, hemocultures but not blood were obtained at different follow-up stages, so qPCR quantification of parasitic loads was not performed.

Overall, 92.06% (116 out of 126 samples) agreement between kDNA-PCR and satDNA-qPCR findings was found (**Supplementary Data**). The percentage of positivity for Pre-Tx samples was 100% (n=25), with a median parasite load of 2.869 [IQR = 2.113 to 3.720] log10 par eq./mL (**Figures 2B, D**). In approximately one-third of patients, parasitic load fluctuations above and below the lower reportable range were detected during follow-up. Despite this, a 92.5% (n=14) cumulative qPCR positivity was obtained up to nine years Post-Tx (**Figure 2B**). During Post-Tx monitoring, a median of the parasite load of 0.105 [IQR = -1.147 to 1.761] log_10_ par eq./mL was obtained, which is close to qPCR detection limit (**Figure 2D**). At 9 years Post-Tx, 50% (n=8) of the samples remained quantifiable by qPCR, with a median of the parasite load of 0.028 [IQR = -0.502 to 1.783] log_10_ par eq./mL. Thus, SatDNA-qPCR was more sensitive than serological analysis to detect treatment failure, allowing detection of 28.57% (n=5) more cases of patients who did not respond to treatment. In sum, treatment failure of around 85% (n=16) was obtained.

**Figure 3:**
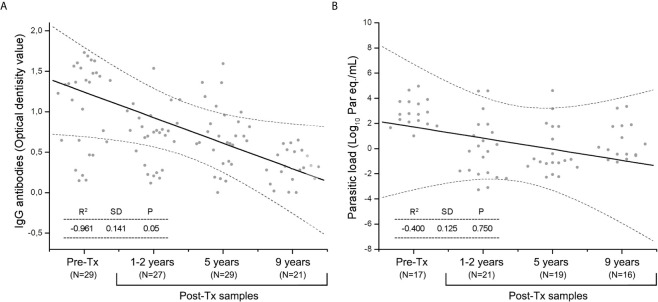
The Spearman’s rank correlation coefficient during Post-Tx follow-up in patients of the Chichiriviche de la Costa oral Chagas disease outbreak. **(A)** IgG serology; **(B)** SatDNA-qPCR parasitic loads. Dotted lines indicate the 95% confidence interval. P value <0.05 indicates the significance of the correlation between the variables.

Serological reactivity decreased during Post Tx follow-up (Spearman’s r value= -0.961, **Figure 3A**), whereas parasitic load showed a slight tendency to decrease at the end of follow-up (Spearman’s r value= -0.400), however, this trend was not statistically significant (P value of the Spearman’s rank correlation coefficient >0.05). Post-Tx parasitic loads showed a median of 0.105 [IQR = -1.147 to 1.761] Log_10_ par.eq/mL along monitoring (**Figure 3B**).

### Characterization of DTUs and Minicircle Signatures

**Figure 4:**
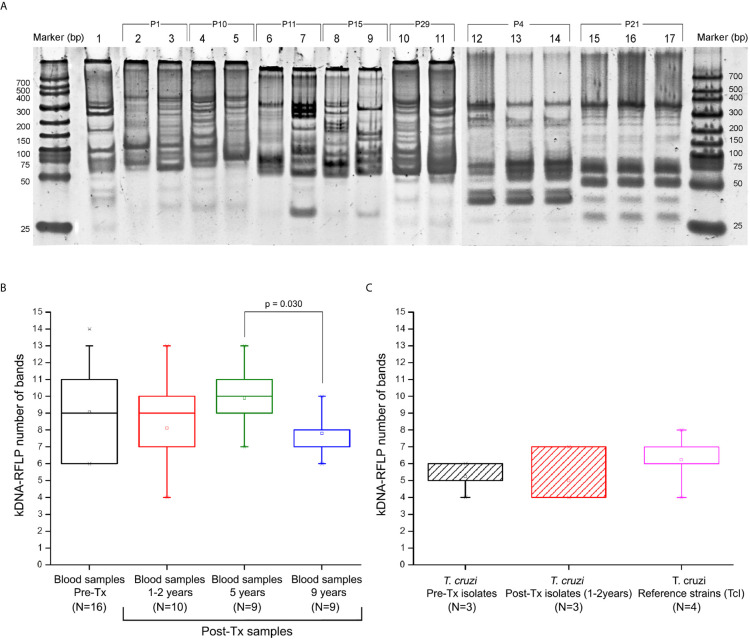
Profile of minicircle signatures in parasite populations by kDNA-PCR restriction fragment polymorphism (RFLP). **(A)** Example of comparative band analysis of minicircle profiles obtained by RFLP-PCR between patients’ parasite populations; **(B)** Boxplot distribution of number bands obtained by RFLP-PCR throughout the follow-up in the blood samples. **(C)** Distribution of the boxplot of number of digestion fragments obtained by RFLP-PCR in *Trypanosoma cruzi* isolates obtained from hemocultures and TcI reference strains. 1: Reference strain (DTU I - Dm28c); 2-3: Sample P1 (Pre-Tx and 5 years Post-Tx); 4-5: Sample P10 (Pre-Tx and 5 years Post-Tx); 6-7: Sample P11 (Pre-Tx and 5 years Post-Tx); 8-9: Sample P15 (Pre-Tx and 5 years Post-Tx); 10-11: Sample P29 (Pre-Tx culture isolate and 1 year Post-Tx culture isolate); 12-14: Sample P4 (Pre-Tx, 5 years and 9 years Post-Tx); 15-17: Sample P21 (Pre-Tx, 5 years and 9 years Post-Tx). The JC results shown in the box “reference strains” were obtained from the comparison of the Ms from the Dm28c, SilvioX10, gal4, and CMA strains. P value indicates significant differences between populations (Mann-Whitney U test; p<0.05). Data in the boxplot are presented as median and interquartile range of 25 to 75%.

Bloodstream DTUs were identified in kDNA-PCR positive samples, resulting all TcI (**Table 1**). The intra-TcI genetic composition was characterized by RFLP-PCR of kDNA amplicons obtained in 41 blood samples from 12 patients at different follow-up time points, and in the hemocultures isolated from 6 patients. The Ms exhibited between five and 14 digestion bands from ~30 to 330 bp of length (**Figure 4A**). A high degree of heterogeneity was observed among different patients` populations and between Pre-Tx and Post-Tx Ms signatures in the same patients.

**Figure 5:**
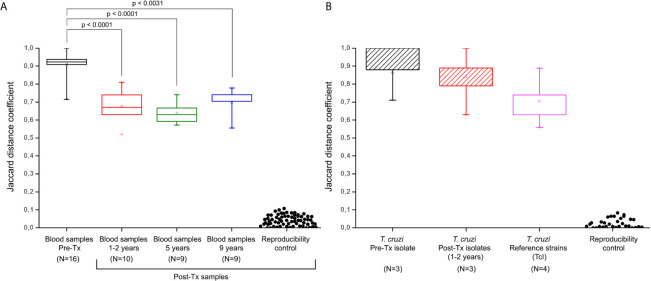
Jaccard distances of minicircle signatures in parasite populations by kDNA-PCR restriction fragment length polymorphism (RFLP). **(A)** Jaccard distances from bloodstream parasites populations in patients of the Chichiriviche de la Costa outbreak. **(B)** Jaccard distances of the hemocultures from the Chichiriviche de la Costa outbreak and TcI reference strains. Reproducibility controls were obtained by comparison of signatures between duplicate samples. Tx: treatment. The JC results shown in the box “reference strains” were obtained from the comparison of the Ms from the Dm28c, SilvioX10, gal4, and CMA strains. P values indicate significant differences between populations (Mann-Whitney U test; p<0.05). Data in the boxplot are presented as median and interquartile range of 25 to 75%.

Comparing the JC values, a slight difference in median values, 0.775 [IQR = 0.708 to 0.882] and 0.857 [IQR = 0.798 to 0.941] were observed in Pre-Tx populations analyzed from clinical samples or culture isolates, respectively (**Figures 5A, B**). However, the Mann-Whitney U test did not show differences between both types of samples. Out of the 25 Post-Tx Ms profiles evaluated, none presented 100% homology with the corresponding Pre-Tx Ms (**Supplementary Data**), revealing that parasite populations changed between the time of diagnosis and times of follow-up. When analyzing the overall *T. cruzi* variability between Pre-Tx and Post-Tx follow-up samples, fluctuations in the degree of polymorphism were observed.

The JC distances decreased respect to Pre-Tx values at 1-2 years and five years Post-Tx (Mann-Whitney U test p<0.0001, respectively). At 9 years Post-Tx, a slight increase in the JC values could be observed compared to those obtained in previous Post-Tx points, however, the difference in values was only significant when comparing the JC values against the Pre-Tx samples (Mann-Whitney U test p= 0.0031). The median of the JC among the three parasite isolates obtained at Pre-Tx was 0.92 and the JC values among the three isolates tested 1-2 years Post-Tx was 0.84 (Mann-Whitney U test p>0.05). Finally, the JC values among the Pre-Tx hemocultures obtained from this outbreak, suggested greater heterogeneity with respect to the JC values obtained in the four TcI reference strains, without reaching significant difference (Mann-Whitney U test p>0.05).

**Figure 6:**
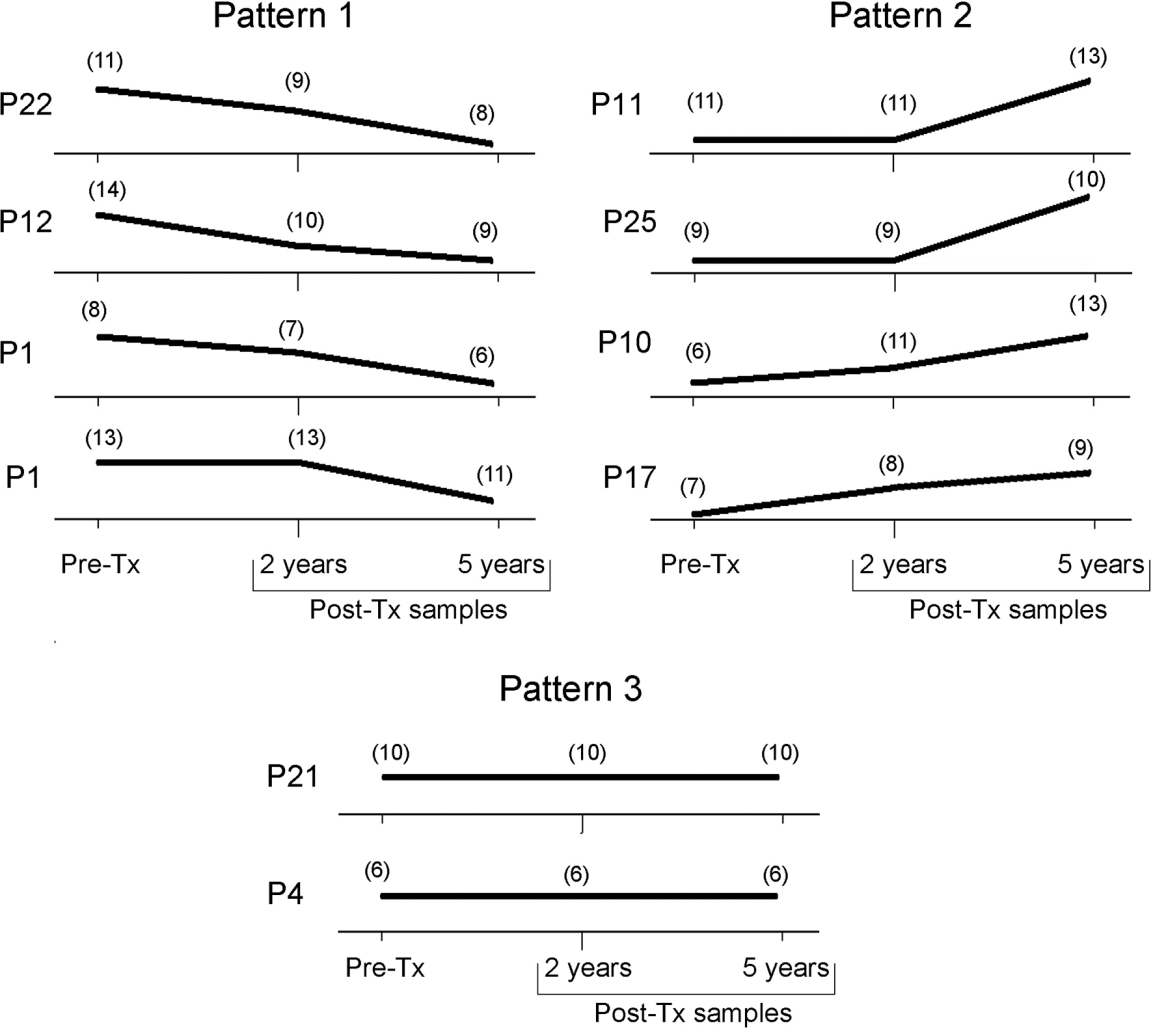
Trend of the genetic variability of natural *T. cruzi* populations by kDNA-RFLP. Each panel represents the count of the number of bands present at each time point along follow-up. Tx, treatment.

The number of Ms restriction fragments from the tested patients’ parasite populations was counted as an indirect measure of the degree of clonality of those populations along follow-up. Different patterns were observed (**Figure 6**). In four patients, the number of Ms bands persisted or showed a slight decrease between Pre-Tx and two years Post-Tx and decreased in complexity when tested five years Post-Tx (pattern 1), suggesting clonal selection. The parasitic populations of other four patients showed an increase in kDNA fragments (pattern 2), which suggests proliferation of a higher number of parasite clones. Finally, two patients (P4 and P21) showed no variation in the number and molecular weights of the Ms restriction fragments of their parasitic populations, except for differences in the intensity of some bands (**Figure 4**).

## Discussion

This is the first study showing the dynamics of parasitic burden and genetic diversity of natural *T. cruzi* populations infecting OCD patients who did not respond to Bnz treatment after nine years of follow-up.

The fluctuations in ELISA-IgG titers and parasitic loads after etiological treatment showed negative Pearson correlation coefficients with a tendency to negativization. Parasite load values at nine years Post-Tx were very close to the lowest value of the qPCR reportable range (**Figure 2D**). However, no seroconversion nor persistent PCR negativization was observed, suggesting a partial parasitological response to Tx, which was even observed in the nine patients who received two rounds of Bnz.

When analyzing parasite loads, the first stands out was the heterogeneity observed in Post-Tx values. This could be associated with the evolution of the infection from its acute phase at time of diagnosis, to the chronic phase during the follow-up period. Low bloodstream parasitic loads are typical of the chronic stage, when parasite persists in tissues according to their clonal histotropism (Macedo and Segatto, 2010). The immune system plays a fundamental role in the control of circulating parasite levels once intracellular amastigotes replicate and differentiate into trypomastigotes that are released to the bloodstream (Macedo and Segatto, 2010), and this can be reflected in the bloodstream qPCR quantifications. Thus, the observed changes may be more related to the passage from one infection phase to the other one than to a selective pressure exerted by chemotherapy, or to a mixture of both factors.

Following the hypothesis proposed by Alarcón de Noya and co-authors, that “*all individuals were infected by a common source for each outbreak, based in eco-epidemiological and molecular genetics studies*” (Alarcon de Noya et al., 2010; Munoz-Calderon et al., 2013; Diaz-Bello et al., 2014; Alarcon de Noya et al., 2016), and identification of TcI DTU in all patients, similarly to what has been detected in other surveys in Venezuela (Carrasco et al., 2012), parasite genetic heterogeneity in patients from Chichiriviche de la Costa was revealed at the intra DTU level and between Pre-Tx and Post-Tx in a same patient. This is indicative of polyclonal populations as sources of oral infection in this outbreak. In fact, in food contaminated with triatomines feces, all metacyclic trypomastigotes present in the intestinal content of the triatomines are involved. It is estimated that a single fecal sample may contain between 3000 to 4000 trypomastigotes per microliter, thus a high clonal complexity may exist (Schaub, 1989).

Wild populations of *T. cruzi* may contain both susceptible and resistant clones to chemotherapeutic drugs, therefore destruction of susceptible forms by drugs leads to the selection and proliferation of resistant subpopulations (Noya et al., 2015). In a subgroup of patients whose parasite populations decreased or persisted in clonal complexity after Tx, suggested by the quantity of minicircle restriction fragments of different lengths, it is tempting to speculate that some drug-driven selection pressure could have played a role in subpopulations selection. (Ms patterns 1 and 3; **Figure 6**). Interestingly, parasite culture isolate from patient P21 harbored a truncated Nitroreductase protein sequence, with a putative role in drug resistance (Unpublished data).

*In vitro* susceptibility studies carried out with *T. cruzi* strains isolated from the patients of the Chacao outbreak showed high heterogeneity in IC_50_ values against Nfx, suggesting that the therapeutic failure could be due in part to a phenotypic variability extant in the original parasite source of oral transmission (Muñoz-Calderón et al., 2019). These findings, together with the high heterogeneity found between Pre-Tx and Post-Tx follow-up bloodstream *T. cruzi* populations of this outbreak are compatible to the existence of drug-driven selective pressure.

On the other side, clonal selection may occur in the natural development of *T. cruzi* infection from its acute to its chronic phase. Indeed, clones capable to escape from the host’s immune system are the ones to proliferate in the chronic phase. The median of the parasitic loads of 0.105 [IQR = -1.147 to 1.761] log10 par eq./mL observed during follow-up are typical of chronic infection. In four patients (P10, P11, P17, and P25) an increase in Ms polymorphism at some point in the Post-Tx follow-up was observed. In general, it is assumed that a higher number of minicircle bands is indicative of a higher level of clonality (Burgos et al., 2010). In all cases, as the JC value with respect to the Pre-Tx Ms is high, it can be hypothesized that the subpopulations proliferating after treatment were hidden into the tissues during treatment. In these particular cases, it could be attributed to possible reinfections, since the area is a region with a risk of active vector transmission (Noya et al., 2015).

A point to be highlighted is that the characterized parasitic populations correspond to bloodstream parasites. However, it is not possible to analyze the genetic diversity of parasites housed in tissues. Sánchez-Valdéz et al. (2018) showed for the first time that *T. cruzi* enters a dormant state, i.e. some amastigotes can interrupt their cellular replication during an *in vitro* infection (Thomas et al., 2018; Alonso-Padilla et al., 2020). This characteristic in bacteria and other protozoa has been related to the recurrence of infection or drug resistance (Sánchez-Valdéz et al., 2018). Therefore, the appearance of different parasite populations in the Post-Tx samples of OCD patients respect to their Pre-Tx populations could also be associated with this feature. Accordingly, we could have been identifying the genetic diversity of intracellular parasite populations that survived in an environment where the drug concentration was lower and they were not necessarily resistant clones. This could be the case for those patients who showed an increase in parasite variability after Tx. On the other hand, dormancy would not be so relevant in TcI strains, at least compared to other DTUs, as reported by Resende et al. (2020).

The limited sample size of the patients for each of the time points evaluated (Pre-Tx, 1-2 years, 5 years and 9 years Post-Tx) has been a study limitation, mainly influenced by the difficulty of extracting parasitic gDNA from blood samples in patients at the chronic phase of Chagas disease. Additionally, the lack of a placebo group limits the analysis of the genetic variability of parasitic populations when evolving from the acute phase to the chronic phase of the disease. The inclusion of a placebo group would have also allowed us to evaluate in greater detail the response of these parasitic populations after the etiological treatment. These limitations must be taken in account when designing future studies. In conclusion, our serological and molecular parasitological findings suggest that synergistic multiple factors, such as the existence of clones with natural drug resistance, selective pressure exerted by trypanomicidal treatment, existence of dormant subpopulations, and evolution of acute oral infection to its chronic phase may account for the persistence of seroreactivity and parasite diversity in this patient cohort after almost a decade of follow-up.

## Data Availability Statement

The original contributions presented in the study are included in the article/**Supplementary Material**. Further inquiries can be directed to the corresponding author.

## Ethics Statement

The studies involving human participants were reviewed and approved by Ethical Review Board of the Instituto de Medicina Tropical “Dr Félix Pifano”-Universidad Central de Venezuela, Caracas, Venezuela (CEC-IMT 019/2010 - December 10, 2010). Written informed consent to participate in this study was provided by the participants’ legal guardian/next of kin.

## Author Contributions 

AM-C and AS were responsible for study design, data analysis, and writing up of the manuscript. ZD-B, BA, and ON-G were responsible for recruitment and clinical care of the patients. AM-C and ZD-B were responsible for the laboratory analysis. AS and BA supervised the molecular and serological related experiments. All authors contributed to the article and approved the submitted version.

## Funding

This work was supported by a Fellowship given by the Programme for biotechnology in Latin America and the Caribbean of the United Nations University (UNU-BIOLAC) [to AM-C], funds from the ANPCyT through PICT 2014-0274 [to AS] and financial support from the Immunology Section - Instituto de Medicina Tropical – Universidad Central de Venezuela.

## Conflict of Interest

The authors declare that the research was conducted in the absence of any commercial or financial relationships that could be construed as a potential conflict of interest.
